# Pathogenic variants in *GNPTAB* and *GNPTG* encoding distinct subunits of GlcNAc-1-phosphotransferase differentially impact bone resorption in patients with mucolipidosis type II and III

**DOI:** 10.1038/s41436-021-01285-9

**Published:** 2021-08-02

**Authors:** Giorgia Di Lorenzo, Lena M. Westermann, Timur A. Yorgan, Julian Stürznickel, Nataniel F. Ludwig, Luise S. Ammer, Anke Baranowsky, Shiva Ahmadi, Elham Pourbarkhordariesfandabadi, Sandra R. Breyer, Tim N. Board, Anne Foster, Jean Mercer, Karen Tylee, Renata Voltolini Velho, Michaela Schweizer, Thomas Renné, Thomas Braulke, Dévora N. Randon, Fernanda Sperb-Ludwig, Louise Lapagesse de Camargo Pinto, Carolina Araujo Moreno, Denise P. Cavalcanti, Michael Amling, Kerstin Kutsche, Dominic Winter, Nicole M. Muschol, Ida V. D. Schwartz, Tim Rolvien, Tatyana Danyukova, Thorsten Schinke, Sandra Pohl

**Affiliations:** 1 Department of Osteology and Biomechanics, University Medical Center Hamburg-Eppendorf, Hamburg, Germany; 2 Post Graduate Program in Genetics and Molecular Biology of Universidade Federal do Rio Grande do Sul, Porto Alegre, Brazil; 3 BRAIN Laboratory, Clinical Experimental Research Center, Hospital de Clinicas de Porto Alegre, Porto Alegre, Brazil; 4 International Center for Lysosomal Disorders, University Medical Center Hamburg-Eppendorf, Hamburg, Germany; 5 Department of Trauma and Orthopedic Surgery, University Medical Center Hamburg-Eppendorf, Hamburg, Germany; 6 Institute for Biochemistry and Molecular Biology, Medical Faculty, University of Bonn, Bonn, Germany; 7 Department of Pediatric Orthopedics, Children’s Hospital Altona, Hamburg, Germany; 8 Department of Orthopedics, University Medical Center Hamburg-Eppendorf, Hamburg, Germany; 9 The Centre for Hip Surgery, Wrightington, Wigan and Leigh NHS Trust, Appley Bridge, Wigan, UK; 10 Manchester University NHS Foundation Trust, Saint Mary’s Hospital, Manchester, UK; 11 Center for Molecular Neurobiology, University Medical Center Hamburg-Eppendorf, Hamburg, Germany; 12 Institute for Clinical Chemistry and Laboratory Medicine, University Medical Center Hamburg-Eppendorf, Hamburg, Germany; 13 Children’s Hospital Joana de Gusmão, Florianópolis, Brazil; 14 Skeletal Dysplasia Group, Department of Medical Genetics, University of Campinas, Campinas, Brazil; 15 Institute of Human Genetics, University Medical Center Hamburg-Eppendorf, Hamburg, Germany; 16 Present Address: Telethon Institute of Genetics and Medicine (TIGEM), Pozzuoli, Napoli Italy

## Abstract

**Purpose:**

Pathogenic variants in *GNPTAB* and *GNPTG*, encoding different subunits of GlcNAc-1-phosphotransferase, cause mucolipidosis (ML) II, MLIII alpha/beta, and MLIII gamma. This study aimed to investigate the cellular and molecular bases underlying skeletal abnormalities in patients with MLII and MLIII.

**Methods:**

We analyzed bone biopsies from patients with MLIII alpha/beta or MLIII gamma by undecalcified histology and histomorphometry. The skeletal status of *Gnptg*^*ko*^ *and Gnptab*-deficient mice was determined and complemented by biochemical analysis of primary *Gnptg*^*ko*^ bone cells. The clinical relevance of the mouse data was underscored by systematic urinary collagen crosslinks quantification in patients with MLII, MLIII alpha/beta, and MLIII gamma.

**Results:**

The analysis of iliac crest biopsies revealed that bone remodeling is impaired in patients with *GNPTAB*-associated MLIII alpha/beta but not with *GNPTG*-associated MLIII gamma. Opposed to *Gnptab*-deficient mice, skeletal remodeling is not affected in *Gnptg*^*ko*^ mice. Most importantly, patients with variants in *GNPTAB* but not in *GNPTG* exhibited increased bone resorption.

**Conclusion:**

The gene-specific impact on bone remodeling in human individuals and in mice proposes distinct molecular functions of the GlcNAc-1-phosphotransferase subunits in bone cells. We therefore appeal for the necessity to classify MLIII based on genetic in addition to clinical criteria to ensure appropriate therapy.

## INTRODUCTION

Bone-forming osteoblasts and bone-resorbing osteoclasts coordinate continuous bone matrix remodeling that is required for healthy and functional bone maintenance. During bone formation, osteoblasts generate the extracellular bone matrix, composed of collagen fibers and proteoglycans, which gradually mineralizes due to the deposition of calcium phosphate crystals [1]. A subset of osteoblasts undergoes terminal differentiation into osteocytes, which form a cellular network within the mineralized bone matrix and regulate bone remodeling and mineral homeostasis [2]. Conversely, for bone resorption, osteoclasts attach tightly to the bone trabecular surface and form the ruffled border, in which an acidic extracellular environment, called resorption lacuna, is generated through the expression of the V-ATPase proton pump and the chloride/proton exchanger CLC-7 [3]. Following acidification, the inorganic part of the bone is dissolved and the organic bone components are degraded by lysosomal enzymes that are released upon exocytosis and fusion of lysosomes with the ruffled border [3, 4].

For insuring proper lysosomal function, newly synthesized lysosomal enzymes are equipped with mannose 6-phosphate (M6P) targeting signals to ensure their receptor-dependent transport from the Golgi apparatus to lysosomes [5]. The GlcNAc-1-phosphotransferase hexameric (α_2_β_2_γ_2_) protein complex is the key enzyme for the formation of M6P residues on large set of lysosomal enzymes in the Golgi apparatus, and is the product of two genes: the *GNPTAB* gene (MIM 607840) encodes the membrane-bound α/β-precursor of the GlcNAc-1-phosphotransferase, which is cleaved by Golgi-resident site-1 protease into the individual α- and β-subunits of the complex, whereas *GNPTG* (MIM 607838) encodes the soluble γ-subunit [6–8]. The mature α- and β-subunits underlie catalytic activity of GlcNAc-1-phosphotransferase toward M6P modification of lysosomal enzymes, while the regulatory γ-subunit enhances this activity [7, 9]. Defective M6P formation results in missorting and hypersecretion of lysosomal enzymes into the extracellular compartment followed by accumulation of various nondegradable macromolecules in dysfunctional lysosomes [5].

Impaired GlcNAc-1-phosphotransferase activity is associated with a subgroup of autosomal recessive lysosomal storage disorders called mucolipidosis (ML) types II and III, whose clinical classification is based on the age of onset and the progression of symptoms [10]. Common major features of the ML diseases are severe skeletal defects, i.e., short stature, bowing of limbs, progressive joint contractures, thoracic deformity, and kyphoscoliosis, radiologically summarized as dysostosis multiplex. Additionally, patients with MLII are characterized by progressive mental and psychomotor retardation, organomegaly, and cardiorespiratory complications leading to death in the first decade of life [11]. Although the clinical onset and manifestations are highly variable in affected individuals, MLIII has been classified as an attenuated form of MLII and is characterized by a later onset and slower disease progression. Moreover, the identification of the disease causing genes in 2000 and 2005 [7, 8] allowed classifying ML into three different types of diseases [12]. According to this revised classification, the severe MLII disease (MIM 252500) is caused by complete inactivation of GlcNAc-1-phosphotransferase due to biallelic variants in *GNPTAB*. The less progressive diseases MLIII alpha/beta (MIM 252600) and MLIII gamma (MIM 252605) are associated with specific biallelic missense variants in *GNPTAB* and all known biallelic variants in *GNPTG*, respectively, each of them being associated with residual GlcNAc-1-phosphotransferase activity [10]. As pathogenic variants in the two disease genes for MLIII, namely *GNPTAB* and *GNPTG*, may cause distinct clinical impact—so far poorly understood—the precise genetic classification is mandatory, not only for confirming the clinical diagnosis but also for the development of potential therapies [13].

To understand the multisystemic pathogenesis of these diseases, mouse models for MLII and MLIII gamma have been generated and studied in the last years. In particular, our skeletal studies revealed growth retardation and low bone mass in MLII mice, which were mostly explained by an unexpected increase in the number of fully active osteoclasts [14, 15]. However, no detailed skeletal analysis was performed in *Gnptg*^*ko*^ mice, the mouse model for MLIII gamma. Nevertheless, *Gnptg*^*ko*^ mice exhibit impaired motor performance and functional joint abnormalities likely associated with fatigue and pain at the joints [16, 17]. So far, no mouse model for the MLIII alpha/beta disease has been described.

In this study, a comprehensive analysis of patients, who were genetically diagnosed and classified as MLII, MLIII alpha/beta or MLIII gamma revealed that pathogenic variants in *GNPTAB* but not *GNPTG* result in increased bone resorption. Consistent with this observation, undecalcified histology and bone-specific histomorphometry in *Gnptg*^*ko*^ mice showed no impaired skeletal remodeling phenotype. Thus, we clearly demonstrate a gene-specific impact on bone remodeling abnormalities in human individuals and in mice, suggesting distinct molecular functions of the GlcNAc-1-phosphotransferase subunits in bone cells.

## MATERIALS AND METHODS

### Patient cohort and genetic diagnostics

This study included a patient cohort consisting of 17 individuals from Europe, Asia, and South America, who were clinically and biochemically diagnosed with MLII or MLIII. For the majority of the patients the clinical and molecular diagnosis was previously described [10, 18–24], while the final molecular diagnoses of patients 1, 2, 8, 9, and 15 were reached within this study. Therefore, polymerase chain reaction (PCR) amplification and Sanger sequencing of the coding exons and adjacent exon–intron boundaries of the *GNPTAB* (NM_024312.4) and *GNPTG* (NM_032520.4) genes were performed using genomic DNA (1, 2, 8, 9, and 15, Table S1). PCR products were directly sequenced using the ABI BigDye Terminator Sequencing Kit (Applied Biosystems, Foster City, CA, USA) and an automated capillary sequencer (ABI 3500; Applied Biosystems). Sequence electropherograms were analyzed using the Sequence Pilot software SeqPatient (JSI Medical Systems, Ettenheim, Germany).

Thereafter, according to the clinical phenotype and the obtained genetic data, patients were classified as MLIII gamma (patients 1 to 7), MLIII alpha/beta (patients 8–14), or MLII (patients 15–17) (Table S1). All 17 individuals were characterized by growth retardation and skeletal abnormalities, which were more pronounced in patients with MLII. Detailed clinical description and genetic diagnosis of the patients 1, 2, 8, 9, and 15 reported here are summarized in the Supplementary Case Reports.

### Human bone biopsy studies

Due to hip dysplasia associated with progressive walking difficulties and hip pain, total hip arthroplasty (THA) was performed in the female patient 1 (MLIII gamma, aged 16 years) and the male patient 8 (MLIII alpha/beta, aged 16 years) (Table S1). The male patient 2 (MLIII gamma, aged 8 years), sibling of patient 1, underwent hip reconstruction surgery to prevent rapid progression of the present hip dysplasia (Table S1). The right femoral head was obtained during THA of patient 1. The resected entire specimen was scanned by high-resolution peripheral quantitative computed tomography (HR-pQCT, XtremeCT^®^, Scanco Medical AG, Brütisellen, Switzerland) at a voxel size of 42 µm, followed by three-dimensional image reconstruction. For further demonstration, a healthy femoral head obtained in the context of a previous study [25] was also imaged by HR-pQCT.

To investigate the bone microstructure and turnover in MLIII patients, diagnostic transiliac crest biopsies were obtained during orthopedic surgery of patients 2 and 8. The iliac crest biopsies were fixed in 3.7% formaldehyde, dehydrated, embedded undecalcified into methylmethacrylate, and subsequently cut into 4-µm-thick sections on a Microtec rotation microtome (Techno-Med GmbH, Munich, Germany) for static histomorphometry. Sections were subjected to toluidine blue staining followed by histomorphometric analysis using the OsteoMeasure system (Osteometrics Inc., Decatur, GA, USA) according to the guidelines of the American Society for Bone and Mineral Research [26] and compared to an age-matched reference cohort from a previous study (Table S2) [27].

### Urinary levels of deoxypyridinoline per creatinine in patients

Deoxypyridinoline (DPD)/creatinine in urine of healthy individuals and patients with MLII, MLIII alpha/beta or MLIII gamma patients was measured using the Immulite and Advia assays, respectively (Siemens Healthineers) and compared to age-matched reference ranges [28, 29].

### Animals

The generation of *Gnptg*^*ko*^ (genetic background C57Bl/6) and *Gnptab*-deficient mice (genetic background [C57Bl/6–129/SvJ, 50:50]) were described previously [9, 15, 17]. *Gnptab*-deficient mice were back-crossed to the pure genetic background C57Bl/6 over six generations. All mice were kept in a pathogen-free environment with a 12-hour light/dark cycle, 45% to 65% relative humidity and 20 °C to 24 °C ambient temperature. The care and use of experimental animals complied with all relevant local animal welfare laws, guidelines, and policies. We generally analyzed littermate mice from heterozygous mating.

### Skeletal analysis of mice

Mice were labeled with two doses of calcein (30 mg/kg, Sigma-Aldrich) 10 and 3 days before sacrifice to evaluate the bone formation rate. After sacrifice the dissected skeletons were fixed in 3.7% PBS-buffered formaldehyde for 18 hours, before they were stored in 80% ethanol. For undecalcified bone histology, the lumbar vertebral bodies L1 to L4 and one tibia of each mouse were dehydrated in ascending alcohol concentrations and then embedded in methylmetacrylate as described previously [14]. Sections of 4-μm thickness were cut in the sagittal plane on a Microtec rotation microtome (Techno-Med GmbH, Bielefeld, Germany). All sections were stained by toluidine blue and von Kossa/van Gieson staining procedures using standard protocols, and histomorphometry was performed using the OsteoMeasure system (Osteometrics Inc., Decatur, GA, USA) according to the guidelines of the American Society for Bone and Mineral Research [15].

### Primary cell analysis

For osteoclast differentiation, bone marrow was flushed out of the femora from 12-week-old mice with α-MEM (Thermo Fisher Scientific) containing 10% fetal bovine serum (FBS). Cells were then plated at a density of 5 × 10^6^ cells per ml, and after 24 hours the adherent cells were cultured in α-MEM containing 10% FBS and 10 nM 1,25-dihydroxyvitamin-D3 (Sigma-Aldrich). Beginning at day 4 after seeding, 20 ng/ml M-Csf (macrophage colony-stimulating factor; Peprotech) and 40 ng/ml Rankl (receptor activator of NF-κB ligand, Peprotech) were added, and the cells were cultured for 7 days to generate osteoclasts. For tartrate-resistant acid phosphatase (TRAP) staining, osteoclasts were washed twice with PBS and fixed with cold methanol for 5 minutes. After two subsequent washes with water, cells were dried for 2 minutes and then stained for 30 minutes using 0.1 mg/ml Naphtol ASMX-Phosphate (Sigma-Aldrich) and 0.6 mg/ml Fast Red Violet (Sigma-Aldrich) as a substrate dissolved in 40 mM sodium acetate, 10 mM sodium tartrate, pH 5.0. For resorption assays, bone marrow cells were seeded onto dentin slices of 1-mm thickness. After 10 days of differentiation, the resorbed areas were visualized and quantified as described [14].

For osteoblast differentiation, bone marrow cells were cultured for 25 days in α-MEM/FBS containing 25 µg/ml ascorbic acid and 5 mM β-glycerophosphate (both from Sigma-Aldrich). Alizarin red staining and quantification of mineralization was performed with alizarin red S solution (40 mM, pH 4.2) followed by dissolving the matrix‐bound alizarin red S in 10% acetic acid and measuring absorbance at 405 nm.

### RNA analysis

Total RNA from primary osteoblasts or osteoclasts of four wild-type and *Gnptg*^*ko*^ mice each was isolated with the PEQ Gold Total RNA Isolation Kit (VWR) according to manufacturer’s instructions. For relative quantitative messenger RNA (mRNA) analysis, RNA isolation from cultured cells, complementary DNA (cDNA) synthesis, and quantitative real-time PCR using predesigned Taqman assays (Thermo Fisher Scientific) was performed as previously described [9]. The relative mRNA level of each analyzed gene was normalized to the level of *Gapdh* mRNA in the same cDNA sample using the comparative CT method (2^–ΔΔCT^).

### Protein analysis

Cells were lysed in PBS containing 0.5% Triton X-100 and protease inhibitors for 30 minutes at 4 °C. After centrifugation at 16,000*g* supernatants were used for measurement of the protein content by the Roti^®^quant Protein Assay (Roth). The enzymatic activities of lysosomal enzymes in protein extracts of primary cultured cells and corresponding media were assayed using corresponding 4-nitrophenol or 4-methylumbelliferone substrates and normalized to total protein content of the lysates [9, 30]. The serum concentration of bone-specific collagen degradation products (C-terminal telopeptides of type I collagen, CTX-I) was determined by enzyme-linked immunosorbent assay (ELISA) (Immunodiagnostic Systems).

Stable isotope labeling by amino acids in cell culture (SILAC) and mass spectrometry analysis are described in detail in the Supplementary Materials and Methods.

### Immunofluorescence and electron microscopy

Primary cultured cells grown on cover slips were fixed with 4% paraformaldehyde in PBS for 30 minutes. After washing with 50 mM ammonium chloride, cells were permeabilized with 0.1% saponine in PBS for 10 minutes and blocked in PBS containing 0.1% saponine and 3% bovine serum albumin for 30 minutes. Subsequently, cells were incubated with primary antibodies against Lamp1 (clone 1D4B, Hybridoma Bank, University of Iowa, USA) for 2 hours. After washing with 0.1% saponine in PBS, cells were incubated with secondary antibodies conjugated to Alexa Fluor^®^ 488 (Thermo Fisher Scientific), and 4′,6-diamidino-2-phenylindole (DAPI) for 1 hours and embedded in Aqua-Poly/Mount. Fluorescence was detected and images were acquired using the FluoView F1000 Olympus digital scanning confocal microscope (Olympus, Tokyo, Japan) and Adobe Photoshop software, respectively.

For ultrastructural analysis, tibiae were fixed with 4% paraformaldehyde and 1% glutaraldehyde in 0.1 M phosphate buffer (pH 7.4) overnight and decalcified for 3 to 4 weeks in 10% EDTA. Thereafter 100-µm-thick vibratome sections were prepared and postfixed in 1% OsO_4_, dehydrated, and embedded in Epon™. Ultrathin sections (60 nm) were cut and mounted on copper grids. Sections were stained using uranyl acetate and lead citrate. Thin sections were examined and photographed using an EM902 (Zeiss) electron microscope equipped with a TRS 2 K digital camera (A. Tröndle, Moorenweis, Germany).

### Statistical analysis

Data are the means ± standard deviations (SD) and significance was evaluated with the unpaired, two-tailed Student’s *t*-test (Microsoft Excel^®^). A result was considered statistically significant if the *P* value was ≤0.05 and the significance was indicated as follows: ≤0.05 (*), ≤0.01 (**) or ≤0.005 (***).

## RESULTS

### Bone remodeling is impaired in patients with MLIII alpha/beta but not with MLIII gamma

**Fig. 1:**
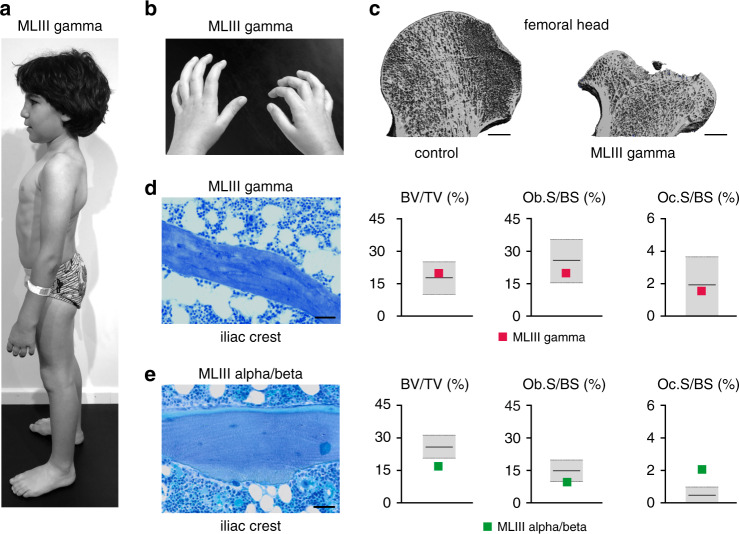
Bone remodeling is not impaired in patients with mucolipidosis III (MLIII) gamma but with MLIII alpha/beta. (**a**) Skeletal manifestation in an 8-year-old male patient with MLIII gamma (patient 2, Table S1). Disabling hip pain and vertebral deformation is the major contributor to morbidity. (**b**) Joint stiffness in patient 2 results in formation of finger flexion contractures. (**c**) Representative coronal views of three-dimensional reconstructions of femoral heads of a healthy control (left panel) and a 16-year-old female patient with MLIII gamma (patient 1, Table S1), indicating severe femoral head osteonecrosis in MLIII gamma. Scale bars: 5 mm. (**d**) Toluidine blue staining of an undecalcified section from a human iliac crest bone biopsy of an 8-year-old male patient with MLIII gamma (patient 2, Table S1). (Scale bar: 50 µm). Quantification of bone volume per tissue volume (BV/TV), osteoblast surface per bone surface (Ob.S/BS) and osteoclast surface per bone surface (Oc.S/BS) of the same MLIII gamma biopsy in relation to the age- and gender-matched reference range (gray boxes) are given on the right. See also Table S2. (**e**) Toluidine blue staining of a undecalcified section from a human iliac crest bone biopsy of a 16-year-old male patient with MLIII alpha/beta (patient 8, Table S1). Scale bar: 50 µm. Quantification of BV/TV, Ob.S/BS, and Oc.S/BS of the same MLIII alpha/beta biopsy in relation to the age- and gender-matched reference range (gray boxes) are given on the right. See also Table S2.

The skeletal status of young children with MLII and adolescents with either MLIII alpha/beta or MLIII gamma has not been fully explored although radiologically the skeletal involvement has been well described as either severe of rather mild dysostosis multiplex. Moreover, genetic discerning two forms of MLIII as nonallelic clinical entities had to await the molecular characterization of the GlcNAc-1-phosphotransferase complex followed by the identification of the genes *GNPTAB* and *GNPTG* encoding its composing proteins [7, 8]. Here we report on our findings in Iraqi siblings (female patient 1 and male patient 2), who presented with skeletal alterations such as growth retardation, joint stiffness, and bone dysplasia fitting within the concept of dysostosis multiplex. The girl underwent bilateral surgical correction of genua valga by temporary epiphysiodesis at 13 years of age, one-sided carpal tunnel syndrome decompression at 14 years of age, and bilateral THA at 16 and 17 years of age. The physical examinations of the patient 2 highlighted joint contractures, especially of the fingers (Fig. 1a, b). At age seven years, skeletal features comprised growth retardation and bilateral hip dysplasia, for which he underwent bilateral hip reconstruction surgery at eight years of age.

Laboratory screening revealed hypersecretion of several lysosomal enzymes into the serum and led to the biochemical diagnosis of MLIII. Genomic sequence analyses of *GNPTAB* and *GNPTG* identified the previously described homozygous pathogenic *GNPTG* variant c.499dup, which brought the specific diagnosis of MLIII gamma in both siblings (Table S1). Painful advancement of hip dysplasia with progressive walking impairment in patient 1 required THA at age 16 years. Significant microarchitectural deformation of the femoral head (Fig. 1c, Fig. S1) measured by HR-QCT precluded reliable histomorphometric evaluation of the skeletal microstructure and bone turnover. Hip reconstruction surgery also required in the younger brother (patient 2) provided the opportunity to study a standardized iliac crest biopsy specimen. Remarkably the quantitative parameters of bone remodeling revealed that the bone volume per tissue volume as well as the osteoblast and osteoclast covered bone surfaces did correspond well to established reference ranges (Fig. 1d, Table S2).

A third, male patient from the United Kingdom (patient 8, Table S1) biochemically and clinically diagnosed in the UK to have MLIII also had progressive joint stiffness and by 13 years of age severe hip pain and much impaired walking ability. Genomic sequence analysis identified the known pathogenic *GNPTAB* variants c.2591_2592insG and c.3335 + 6T>G [10] with the clinical implication of MLIII alpha/beta as formal diagnosis. He presented early and progressive joint stiffness. By 13 years of age, he had considerable hip pain and his walking became limited. At age of 16 years, he underwent bilateral THA and also offered the opportunity for an iliac crest biopsy. Histomorphometric study of the specimen revealed here decreased bone volume per tissue volume, and cellular histomorphometry identified a pathological increase in the osteoclast surface per bone surface (Fig. 1e, Table S2). The data point at impaired bone remodeling in this patient 8 with MLIII alpha/beta, which was not found in patient 2 with MLIII gamma.

### Moderate lysosomal dysfunction in bone cells does not affect bone mass and bone formation in *Gnptg*^*ko*^ mice

**Fig. 2:**
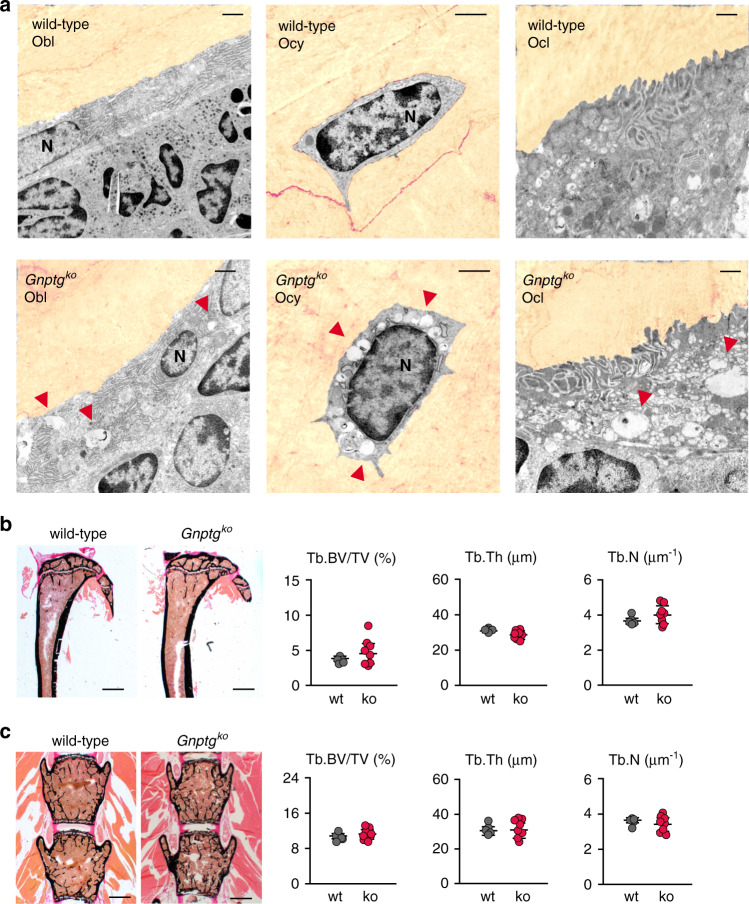
Lysosomal storage accumulation in bone cells does not impair bone microstructures in *Gnptg*^*ko*^ mice. (**a**) Ultrastructural analysis of decalcified tibiae cryosections from 25-week-old wild-type and *Gnptg*^*ko*^ mice. The bone matrix is pseudocolored (sepia). Note the electron-lucent enlarged lysosomes (red arrowheads) in osteoblasts (Obl), osteocytes (Ocy) and osteoclasts (Ocl). N nuclei. Scale bars: 1 µm. (**b**) Representative von Kossa/van Gieson staining of undecalcified tibia sections from wild-type (wt) and *Gnptg*^*ko*^ (ko) mice. Scale bars: 1 mm. Quantification of the trabecular bone volume per tissue volume (Tb.BV/TV), trabecular thickness (Tb.Th) and trabecular number (Tb.N) from the same mice are given on the right (wt, *n* = 5; ko, *n* = 8; mean ± SD). (**c**) Representative von Kossa/van Gieson staining of undecalcified vertebra sections from 25-week-old wt and *Gnptg*^*ko*^ mice. Scale bars: 1 mm. Quantification of Tb.BV/TV, Tb.Th, and Tb.N from the same mice are given on the right (wt, *n* = 5; ko, *n* = 8; mean ± SD).

Recently, we have shown that *Gnptg*-dependent mistargeting of lysosomal enzymes results in accumulation of lysosomal storage material in chondrocytes and tenocytes, which impairs the functionalities of the cartilage and the Achilles tendon [17]. Similarly, by electron microscopy of ultrathin tibial bone sections a few electron-lucent vacuolar structures were observed in osteoblasts, terminally differentiated osteocytes, and osteoclasts from *Gnptg*^*ko*^ mice (Fig. 2a).

Consistent with our findings in the patient 2 with *GNPTG*-associated MLIII gamma, no significant differences in trabecular bone parameters were observed at either 25 or 45 weeks of age between *Gnptg*^*ko*^ and wild-type control mice in undecalcified tibiae or spine (Fig. 2b, c and Fig. S2a). In contrast, strongly decreased bone mass was detectable in 12-week-old *Gnptab*^*−/−*^ mice, the mouse model for MLII (Fig. S3).

**Fig. 3:**
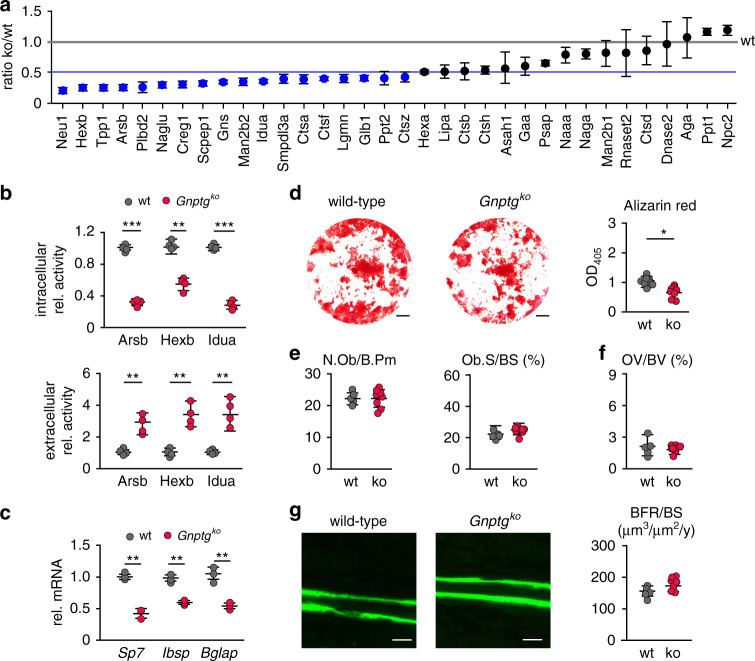
Impaired M6P formations on lysosomal enzymes in *Gnptg*^*ko*^ osteoblasts does not compromise bone formation in *Gnptg*^*ko*^ mice. (**a**) Lysosomal enzymes from wild-type (wt) and *Gnptg*^*ko*^ osteoblasts identified by stable isotope labeling by amino acids in cell culture (SILAC) followed by M6P affinity chromatography and mass spectrometry analysis are displayed as ko/wt ratio (experimental details can be found in the Supplementary Materials and Methods). Significantly reduced amounts (<50%, blue line) of lysosomal enzymes in *Gnptg*^*ko*^ samples are indicated by blue circles (*n* = 3 mean ± SD). The abbreviations of all proteins correspond to their respective gene names. (**b**) Relative enzyme activities of arylsulfatase B (Arsb), β-hexosaminidase (Hexb) and α-iduronidase (Idua) in cell extracts and corresponding conditioned media of wt and *Gnptg*^*ko*^ osteoblasts (*n* = 4, mean ± SD, **P ≤ 0.01, ***P ≤ 0.005). (**c**) Relative messenger RNA (mRNA) expression levels of the transcription factor *Sp7*, bone sialoprotein (*Ibsp*), and osteocalcin (*Bglap*) in wt and *Gnptg*^*ko*^ osteoblasts (*n* = 3, mean ± SD, **P ≤ 0.01). (**d**) Representative Alizarin red staining of primary osteoblasts from wt and *Gnptg*^*ko*^ mice. Scale bars: 0.25 cm. Quantification of Alizarin red incorporation is given on the right (*n* = 8, mean ± SD, *P ≤ 0.05). (**e**) Quantification of cellular parameters in vertebra sections from 25-week-old wt and *Gnptg*^*ko*^ (ko) mice: number of osteoblasts per bone perimeter (N.Ob/B.Pm), osteoblast surface per bone surface (Ob.S/BS) (*n* = 4, mean ± SD). (**f**) Quantification of the vertebral osteoid volume per bone volume (OV/BV) in wt and *Gnptg*^*ko*^ (ko) mice (wt, *n* = 5; ko, *n* = 8; mean ± SD). (**g**) Representative calcein-labeled bone surfaces of vertebral bodies from 25-week-old wt and *Gnptg*^*ko*^ (ko) mice. Scale bars: 10 µm. Quantification of the bone formation rate per bone surface (BFR/BS) of the same mice is given on the right (wt, *n* = 5; ko, *n* = 8; mean ± SD).

To investigate which lysosomal enzymes in osteoblasts require the presence of the γ-subunit for efficient M6P modification in wild-type and *Gnptg*^*ko*^ mice SILAC followed by M6P affinity chromatography and mass spectrometry was performed [9]. We identified 34 lysosomal enzymes and quantified them based on the *Gnptg*^*ko*^/wild-type ratio (Fig. 3a; Table S3). Among those, a significant reduction to ≤50% of the wild-type levels was observed for 18 lysosomal enzymes involved in the lysosomal degradation of glycans (e.g., Neu1, Hexb, Arsb, Gns, Man2b2, Idua, and Glb1), proteins (e.g., Tpp1, Ctsa, Ctsf, and Ctsz) and lipids (Plbd2 and Ppt2). Of note, the reduced protein amounts were not caused by decreased expression of mRNA expression of these lysosomal enzymes (Fig. S4). In agreement with the proteomic data, decreased intracellular and increased extracellular activities of the lysosomal enzymes Arsb (arylsulfatase B), Hexb (β-hexosaminidase), and Idua (α-iduronidase) were found in cultured *Gnptg*^*ko*^ osteoblasts compared to wild-type cells (Fig. 3b). These results show that the γ-subunits in the GlcNAc-1-phosphotransferase complex are required for efficient targeting of some lysosomal enzymes in osteoblasts.

By assessing the function of primary *Gnptg*^*ko*^ osteoblasts, we observed lower mRNA levels of the genes *Sp7* (transcription factor Sp7)*, Ibsp* (bone sialoprotein), and *Bglap* (osteocalcin) involved in osteoblast differentiation and bone formation in *Gnptg*^*ko*^ compared to mRNA levels in wild-type osteoblasts (Fig. 3c). This finding was complemented by a significant reduction of mineralization as demonstrated by Alizarin red staining (Fig. 3d). Cellular histomorphometry on vertebral body sections from *Gnptg*^*ko*^ mice revealed that neither osteoblast numbers nor covered bone surfaces (Fig. 3e, S2a) differed from the findings in the wild-type controls. There was no increased amount of nonmineralized osteoid per bone volume in *Gnptg*^*ko*^ mice (Fig. 3f, S2b). To measure the rate of bone formation, additionally dynamic histomorphometry was applied after dual calcein injections into mice. Here again no significant differences were observed between wild-type and *Gnptg*^*ko*^ mice in the rate of bone formation (Fig. 3g).

### Bone resorption is not demonstrably affected in *Gnptg*^*ko*^ mice

**Fig. 4:**
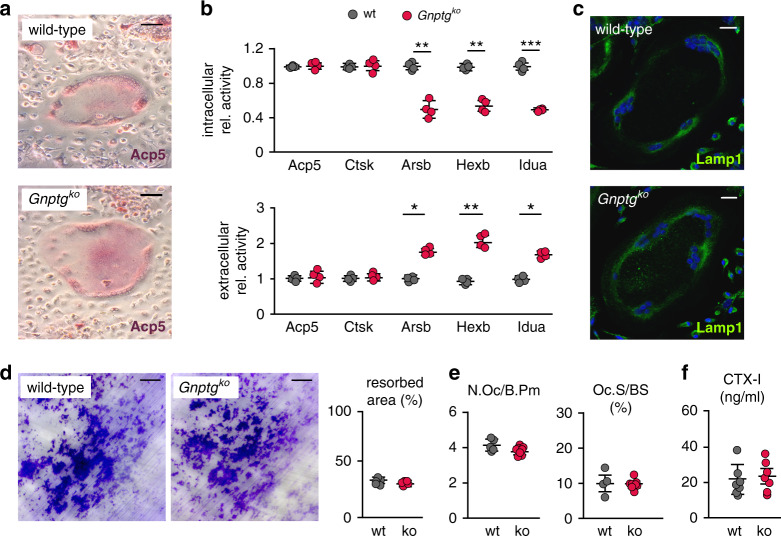
Moderately impaired lysosomal maintenance of *Gnptg*^*ko*^ osteoclasts does not affect bone resorption in 25-week-old *Gnptg*^*ko*^ mice. (**a**) Representative Acp5 (also known as tartrate-resistant acid phosphatase, TRAP) activity staining of wild-type (wt) and *Gnptg*^*ko*^ osteoclasts. Scale bars: 20 µm. (**b**) Relative enzyme activities of Acp5, cathepsin K (Ctsk), arylsulfatase B (Arsb), β-hexosaminidase (Hexb), and α-iduronidase (Idua) in cell extracts and corresponding media of cultured wt and *Gnptg*^*ko*^ osteoclasts (*n* = 4, mean ± SD, *P ≤ 0.05, **P ≤ 0.01, ***P ≤ 0.005). (**c**) Representative immunofluorescence staining of wild-type and *Gnptg*^*ko*^ osteoclasts using an antibody against Lamp1. Nuclei are stained with DAPI (blue). Scale bars: 20 µm. (**d**) Quantification of toluidine blue-stained area covered by resorption pits after 10 days cultivation of osteoclasts on dentine chips (*n* = 5, mean ± SD). Scale bars = 50 µm. (**e**) Quantification of cellular parameters in vertebra sections from wt and *Gnptg*^*ko*^ (ko) mice: number of osteoclasts per bone perimeter (N.Oc/B.Pm), osteoclast surface per bone surface (Oc.S/BS) (wt, *n* = 5; ko, *n* = 8; mean ± SD). (**f**) C-terminal telopeptides of type I collagen (CTX-I) in wt and *Gnptg*^*ko*^ (ko) mice (*n* = 6; mean ± SD).

Bone resorption strongly depends on the action of the lysosomal enzymes cathepsin K (Ctsk) and tartrate-resistant acid phosphatase 5 (Acp5, TRAP), which are established osteoclast differentiation markers. Whereas Acp5 is required for dephosphorylation of M6P targeting signals in osteoclasts to promote the activity of specific lysosomal enzymes, Ctsk is directly involved in the degradation of bone matrix collagens [4, 30]. However, we found that the activity of Acp5 and Ctsk remained undisturbed in *Gnptg*^*ko*^ osteoclasts (Fig. 4a, b). To the contrary, the activity of the lysosomal enzyme arylsulfatase B (Arsb), recently found to be an additional key player in bone resorption [30, 31], was decreased intracellularly and increased extracellularly in *Gnptg*^*ko*^ osteoclasts compared to wild-type cultures (Fig. 4b). Similar missorting was observed for the lysosomal enzymes β-hexosaminidase (Hexb) and α-iduronidase (Idua) (Fig. 4b). Only a small number of enlarged lysosomes was observed in *Gnptg*^*ko*^ osteoclasts in vivo by electron microscopy (Fig. 2a), but the immunofluorescence signal intensity of Lamp1-positive lysosomes in primary cultured *Gnptg*^*ko*^ osteoclasts was comparable to that in wild-type cells (Fig. 4c). Most importantly, the resorption efficiency of *Gnptg*^*ko*^ osteoclasts was similar to wild-type cells (Fig. 4d). Consistently, no difference in osteoclast numbers and covered bone surfaces, assessed by cellular histomorphometry on vertebral body sections, between wild-type and *Gnptg*^*ko*^ mice was observed either (Fig. 4e, S2a). Finally, the serum quantity of the bone resorption biomarker CTX-I does not support excessive bone resorption in 25- or 45-week-old *Gnptg*^*ko*^ mice (Fig. 4e, S2c).

### Increased bone resorption in ML patients is caused by variants in *GNPTAB*, but not in *GNPTG*

The lack of bone remodeling defects in *Gnptg*^*ko*^ mice was confirmed by the normal serum concentration serum of the bone resorption biomarker CTX-I (C-terminal telopeptides of type I collagen), as had also been found in *Gnptab*-deficient MLII mice [14, 15]. Likewise, increased urinary levels of deoxypyridinoline (Dpd) derived from bone collagen resorption were measured in three unrelated infants with MLII [14, 32, 33].

**Fig. 5:**
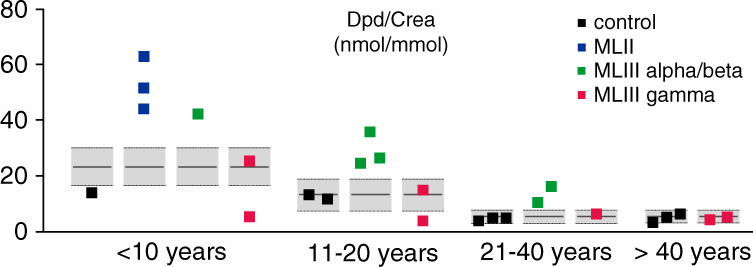
Bone resorption is increased in patients with mucolipidosis (ML) II and MLIII alpha/beta but not MLIII gamma. Urinary desoxypyridinoline/creatinine ratio (Dpd/Crea) from patients with MLII, MLIII alpha/beta, and MLIII gamma, as well as from control individuals in relation to the age-matched reference range (gray boxes) [28, 29]. Of note, due to the premature death of patients with MLII and MLIII alpha/beta those patients are not present in all cohorts.

Determination of collagen degradation molecules, likely to be helpful in relating excessive bone resorption to either *GNPTAB* or *GNPTG* variant genotypes, was performed in the urine samples collected from 17 patients with the diagnosis of MLII, MLIII alpha/beta and MLIII gamma and in 9 age-matched healthy individuals and compared with published reference data [29] (Table S1). As expected, the urinary levels of Dpd normalized to creatinine (Dpd/Crea) decreased with age due to enhanced bone remodeling during skeletal development, with very high values in childhood and lower levels in adulthood (Fig. 5). All three patients with MLII aged 0.7 to 4 years displayed excessive bone resorption. Of note, the short clinical course and premature death of these patients precludes laboratory values for later ages. Importantly, Dpd/Crea ratios in urinary samples from all six patients with MLIII alpha/beta aged 6 to 34 years was consistently higher and indicates more excessive bone resorption. In sharp contrast, none of the individuals with MLIII gamma aged 6 to 49 years showed rather similar urinary Dpd/Crea levels to control individuals. Of note, the lower values in the older adults may have been higher at a younger age, when bone metabolism and turnover processes are more intense. This key observation demonstrates that patients with *GNPTAB*-associated MLII and MLIII alpha/beta, but not *GNPTG*-associated MLIII gamma, exhibit increased bone resorption. These findings are in line with the probably ancillary role of the γ-subunits in the GlcNAc-1-phosphotransferase complex in bone tissue.

## DISCUSSION

In this study, we convincingly show that in the absence of the *Gnptg*-encoded γ-subunit of the GlcNAc-1-phosphotransferase lysosomes in osteoblasts and osteoclasts endure moderate dysfunction due to deficient lysosomal delivery of a large subset of lysosomal enzymes. However, in contrast to *Gnptab*-deficient mice [14, 15], deep skeletal phenotyping revealed no impaired bone remodeling in *Gnptg*^*ko*^ mice. Most importantly, the comprehensive patient analysis provided evidence that patients with *GNPTAB*-associated MLII and MLIII alpha/beta, but not with *GNPTG*-associated MLIII gamma, exhibit increased bone resorption, which strongly corroborates our in vivo and in vitro results obtained in the respective mouse models.

In previous studies using two different mouse models of MLII, we have identified impaired bone formation and increased osteoclastogenesis causative for the *GNPTAB*-associated bone pathology in MLII mice [14, 15]. In accordance with these findings, increased bone resorption was detected in patients with MLII [14, 32, 33]. Although murine MLII osteoclasts were not functionally impaired, osteoclastogenesis was increased in MLII mice. Considering the MLII phenotype correction through antiresorptive bisphosphonate treatment, we therefore concluded that excessive osteoclastogenesis is the major driver of MLII-associated bone loss [14] and raised the question whether excessive bone resorption is one of the complications in patients with MLIII as well. In fact, a positive effect of bisphosphonate treatment was described for some patients with MLIII [34, 35], highlighting the relevance of studying skeletal phenotype using a mouse model of MLIII, as achieved in the present study.

Here, the *Gnptg*^*ko*^ mice underwent the same detailed skeletal analyses as we have applied to *Gnptab*-deficient mice [14, 15] (Fig. S2). Thereby we could not detect skeletal abnormalities in *Gnptg*^*ko*^ mice. In contrast to impaired differentiation of *Gnptab*-deficient osteoblasts, *Gnptg*^*ko*^ bone cells displayed a less pronounced pathology and therefore did not significantly impact the bone formation rate. Of note, the dramatic failure of primary MLII osteoblasts to produce a mineralized matrix as well as strongly decreased expression of osteogenic marker genes in vitro was also only partially reproduced in vivo, as evidenced by a less than 30% reduction of the bone formation rate [14]. Most importantly however, no increased osteoclastogenesis was observed in *Gnptg*^*ko*^ mice, and the serum levels of the bone resorption biomarker CTX-I did not differ from wild-type controls.

To address the question whether similar differences can be observed in distinct forms of MLIII in humans, we analyzed bone biopsies from patients with genetically confirmed MLIII alpha/beta (*GNPTAB* variants) and MLIII gamma (*GNPTG* variants). In particular, patient 2 and patient 8 (Table S1) with confirmed diagnosis of MLIII gamma or MLIII alpha/beta, respectively, required total hip arthroplasties to remedy painful and impaired walking. Importantly, no abnormal bone resorption features could be demonstrated in the iliac crest biopsy specimens of the patient with MLIII gamma. To the contrary, the same advanced skeletal analysis of a corresponding bone specimen from the patient with MLIII alpha/beta increased bone resorption was detected. Hence, our results have clearly confirmed, that MLIII alpha/beta and MLIII gamma are not only genetically but also phenotypically different. Although the genetic bases of both diseases are easily detectable, the clinical entities remain hardly discernible as patients present with similar signs, symptoms, and clinical course and radiographically with congruent dysostosis multiplex [10, 12]. This issue has been recently addressed [36] and deserves more studies in the future.

This important conclusion is supported by comparing the Dpd levels (normalized to creatinine) in the urine samples of the genetic subgroups of ML patients represented in the heterogeneous cohort of 17 patients (Table. S1). Although these diseases are ultrarare, with approximately 100 patients with MLIII alpha/beta and less than 40 patients with MLIII gamma reported worldwide to date [10], the impact of variants in the different genes was strikingly obvious in our cohort. Specifically, urinary Dpd levels were pathologically increased in all individuals with biallelic *GNPTAB* variants compared to age-matched reference ranges, but remained within the reference ranges in all individuals with biallelic *GNPTG* variants. It may be pointed out that the variant *GNPTAB* c.10A>C of patient 10 (Table S1) causes an intermediate MLII/MLIII alpha/beta phenotype [10, 11, 37], which combines clinical features of MLII, such as skeletal alterations and growth impairment with only minor intellectual disability and life expectancy as in MLIII. It illustrates that the mucolipidosis types II, III alpha/beta and III gamma are multisystem disorders and that the composing subunits in the GlcNAc-1-phosphotransferase complex may matter differently in various tissues. Particularly, the collective data of this study demonstrate that *GNPTG* gene defects do not cause the same bone remodeling abnormalities as *GNPTAB* variants, which is an important observation, as effective treatment options to counteract osteoclast-mediated bone loss in MLIII alpha/beta patients are in principle available.

The γ-subunit of GlcNAc-1-phosphotransferase enhances the activity of the whole enzyme complex for a subset of lysosomal enzymes in different cell types [9, 38–40]. We have shown here that in bone cells, similar to fibroblasts and chondrocytes, the efficient intracellular targeting of lysosomal enzymes involved in the degradation of glycosaminoglycans depends on the presence of the γ-subunit. In fact, sulfated glycosaminoglycans are present in proteoglycans, which are highly secreted by osteoblasts and chondrocytes to generate the extracellular matrix of bones and cartilage, respectively [41]. However, in contrast to chondrocyte differentiation and cartilage homeostasis compromised by lysosomal dysfunction in *Gnptg*^*ko*^ mice [17], the bone cell function is maintained by the amounts of lysosomal enzymes that are still targeted to lysosomes in the absence of the γ-subunit. Since joint stiffness has been recognized as a hallmark of MLIII gamma [10], we conclude that cartilage and subchondral bone defects as well as functional abnormalities of the joint are the major dysfunctions of MLIII gamma, while alterations in bone remodeling are characteristic features of MLII and MLIII alpha/beta. Based on these findings, we postulate that molecular functions of the GlcNAc-1-phosphotransferase subunits are distinct in bone cells and need to be studied in the future. To this end, the generation of a mouse model for MLIII alpha/beta would be particularly supportive.

Studying the skeletal and growth abnormalities in humans remains challenging because of the low incidence of the mucolipidosis diseases and even more because of the low life expectancy in the most severely affected patients with MLII. Our study provides an excellent example of the necessity for genotype–phenotype relationships to fully understand this most specific morbid consequences of the pathogenesis. This is particularly required in the case of MLIII, since the gene harboring pathogenic variants (*GNPTAB* vs. *GNPTG*) is likely to influence not only the formation of osteoclasts, but also the functionality of other cell types. Moreover, from a therapeutic perspective, it is conceivable that patients with MLIII alpha/beta might benefit from antiresorptive treatment more than those with MLIII gamma. Considering that bisphosphonates should not be administered for long time periods, especially in growing individuals [42], the specific knowledge about the type of MLIII, either alpha/beta or gamma, provides a basis for appropriate and evidence-based therapy.

## Supplementary information

Supplementary Information

## Data Availability

All raw and processed data are available upon request.
