# Supplementary material for: Pathogenic variants in GNPTAB and GNPTG encoding distinct subunits of GlcNAc-1-phosphotransferase differentially impact bone resorption in patients with mucolipidosis type II and III
Source: Genet Med. 2021 Aug 2;23(12):2369–77. doi: 10.1038/s41436-021-01285-9 (PMC8629757; doi:10.1038/s41436-021-01285-9)
Supplement: Supplementary file 1 — Supplementary Information [file 41436_2021_1285_MOESM1_ESM.pdf]

SUPPLEMENTARY INFORMATION

Pathogenic variants in *GNPTAB* and *GNPTG* encoding distinct subunits of GlcNAc-1-phosphotransferase differentially impact bone resorption in patients with mucolipidosis type II and III

Di Lorenzo G, Westermann LM, Yorgan TA, Stürznickel J, Ludwig NF, Ammer LS, Baranowsky A, Ahmadi S, Pourbarkhordariesfandabadi E, Breyer SR, Board TN, Foster A, Mercer J, Tylee K, Velho RV, Schweizer M, Renné T, Braulke T, Randon DN, Sperb-Ludwig F, Lapagesse de Camargo Pinto L, Moreno CA, Cavalcanti DP, Amling M, Kutsche K, Winter D, Muschol NM, Schwartz IVD, Rolvien T, Danyukova T, Schinke T, Pohl S

- Supplementary Case Reports: pages 2-4
- Supplementary Materials and Methods: pages 5
- Supplementary Figures: pages 6-7
- Supplementary Tables: pages 9-10
- Supplementary References: pages 11-12

SUPPLEMENTARY CASE REPORTS

Patients #1 and #2 (MLIII gamma)

The siblings from consanguineous healthy Iraqi parents were born spontaneously at term after uneventful pregnancies. Postnatal neurocognitive and motor development of the older sister (patient #1) was unremarkable until progressive joint stiffness was noted at 4 years of age. Biochemical work-up revealed elevated levels of several lysosomal enzymes in plasma, suggesting the diagnosis of MLII/III. She underwent bilateral surgical correction of genua valga by temporary epiphysiodesis at 13 years of age, one-sided carpal tunnel syndrome decompression at 14 years of age and bilateral total hip arthroplasty (THA) at 16 and 17 years of age. At age 17 years, she showed growth retardation and vertebral formation defects (i.e. chondrodystrophic changes, no eminent kyphoscoliosis) suggestive of *dysostosis multiplex*.

The postnatal physical examinations of the younger brother (patient 2) highlighted joint contractures, especially of the fingers. Bilateral carpal tunnel syndromes were released at 5 years of age. At age seven years, skeletal features comprised growth retardation and bilateral hip dysplasia, for which he underwent bilateral hip reconstruction surgery at eight years of age. An iliac crest biopsy was taken at this point.

At last follow-up at 17 and seven years of age, both siblings presented with mildly coarsened facial features. Besides mild aortic valve insufficiency and corneal clouding in the older sister and hyperopia in the younger brother, no other pathologies were noted. They showed normal intellectual functioning and no evidence of cord compression.

Genetic analysis of *GNPTAB* and *GNPTG* by Sanger sequencing identified the homozygous *GNPTG* variant c.499dup in both affected individuals, which led to the classification of MLIII gamma. The frameshift variant c.499dup (p.Leu167Profs*32) was previously identified in two families from Israel and Turkey.[1-3]

Patient #8 (MLIII alpha/beta)

The male patient from the UK was born following a normal pregnancy. The newborn presented no abnormal features besides a deep sacral dimple and positional talipes. At one year of age, joint stiffness was noticed, which was treated with splints. At the age of seven years, mitral regurgitation was diagnosed. At this age he did not show any signs of growth disturbance and had normal intellectual functioning by clinical impression (no formal neurocognitive testing performed). By eight years of age, he had developed an abnormal tooth alignment and the joint contractures had progressed. He underwent temporary epiphysodesis for genua valga and carpal

tunnel syndrome was bilaterally decompressed at the age of nine years. By 13 years of age, because of hip dysplasia, he suffered from severe hip pain affecting his mobility. Consecutively, bilateral endoprothetic total hip replacements were conducted at 16 years of age and iliac crest biopsy was taken at this point. After postoperative recovery, he could walk independently with an Oxford Hip Score of 10/48 (48 worst score, 0 best possible) two years after surgery.

Sanger sequencing revealed the well-known pathogenic *GNPTAB* variants c.2591_2592insG (p.Asn865Lysfs*3)[3,4] and c.3335 + 6T>G (p.Phe1084Valfs*2)[3-8] in heterozygosity, which fits in the clinical diagnosis of MLIII alpha/beta.

Patient #9 (MLIII alpha/beta)

The male patient born from healthy non-consanguineous Brazilian parents was born after an uneventful pregnancy. Besides a tiptoe walking behaviour, which was treated with suropodalic orthosis, the patient showed a normal neurocognitive development at the end of the first year.

At six years of age, myopia was diagnosed and radiological examination revealed *dysostosis multiplex* with alterations in the cervical spine, hands, hips and feet. Subsequent testing of lysosomal enzymes showed elevated lysosomal enzyme levels in the plasma, suggestive of MLII/III. At seven years of age, he underwent surgery for mastoiditis. At last follow-up at the age of nine years, he showed mild hearing loss, pectus carinatum and joint contractures. Finger flexion contractures caused writing difficulties, but he had normal neurocognitive development.

Genetic analysis of *GNTPAB* by Sanger sequencing revealed the variants c.1196C>T (p.Ser399Phe) and c.2757_2758del (p.Asp919Glufs*21), which supported the clinical diagnosis of MLIII alpha/beta. Whereas the missense variant c.1196C>T was found in patients world-wide[3-5,9-12], the frameshift variant c.2757_2758del is novel and was identified in heterozygosity in the mother whereas no DNA was available from the father.

The novel variant has been submitted to the Leiden Open Variant Database (LOVD): https://databases.lovd.nl/shared/configuration/GNPTAB

Patient #15 (MLII)

The male patient was the second child from healthy non-consanguineous Brazilian parents. The previous child was most probably also affected as it had a narrow chest and crooked feet and died 19 days after birth without a diagnosis. A prenatal ultrasound examination of the patient #15 at 23 weeks of gestation had indicated micromelia (small fetus with shortened long bones).

The patient was born by Caesarean section at 36 weeks of gestation. In the neonatal period, he showed respiratory distress which was treated with mechanical ventilation. Hospital discharge occurred at two months of age, still dependant on supplemental oxygen insufflations.

The physical examination at seven months of age highlighted growth retardation, shortened limbs with excessive skin folds, muscular hypotonia and facial dysmorphia with coarsening, arched eyebrows, proptosis, epicantal folds, gingival hypertrophy. The echocardiogram showed a mild tricuspid insufficiency and the abdominal ultrasound hepatomegaly. Joint contractures involved ulnar deviation, deep furrows in the palms and overlapping toes. Radiological evaluations revealed *dysostosis multiplex* with a decreased bone mineralization.

At follow-up, the child presented with neurocognitive delay, respiratory insufficiency and a record of frequent hospitalizations due to recurrent respiratory infections. He died before the age of 24 months of age.

Biochemical work-up revealed elevated levels of several lysosomal enzymes in plasma. Subsequent analysis of *GNPTAB* by Sanger sequencing identified the known homozygous frameshift variant c.2249dup (p.Asn750Lysfs*8), which was previously found in heterozygosity in a Finish patient with MLII (c.1581del/c.2249dup)[3,11] and in two unrelated Brazilian patients with MLII (c.3503_3504del/c.2249dup)[3,13] and MLIII alpha/beta (c.242G>T/c.2249dup)[3,13].

SUPPLEMENTARY MATERIALS AND METHODS

Stable isotope labeling by amino acids in cell culture (SILAC) and mass spectrometry

Gnptg^{ko} (heavy isotope-labeled, Arg$^{13}C_6$ $^{15}N_4$, Lys$^{13}C_6$ $^{15}N_2$) and wild-type (light isotope-labeled) cells were cultured for 24 hours in SILAC-DMEM (Thermo Fisher Scientific) supplemented with 10 mM NH_4Cl. Collected media were concentrated and M6P-containing proteins enriched from 250 µg protein starting material using a single-chain M6P antibody fragment immobilized to AminoLink Plus Gel beads as described elsewhere.[16] Proteins bound to M6P beads were on-bead digested as described previously.[16] Briefly, proteins were reduced and alkylated using dithiothreitol and acrylamide, 5 µg trypsin were added and digestion was performed overnight at 37°C. The next day, the supernatant was transferred to a new tube, the beads were extracted twice and all solutions were pooled. Sample volumes were concentrated using a vacuum centrifuge, desalted using STAGE tips,[17] dried using a vacuum centrifuge, and resuspended in 20 µl 5% acetonitril, 5% formic acid. Samples were analyzed by nanoUHPLC-MSMS using an Easy-nLC 1000 in combination with an Orbitrap Velos (both Thermo Fisher Scientific, Waltham, MA, USA). Peptides were directly loaded on in-house manufactured analytical columns (for details see Ref[16]) and eluted with a linear gradient from 100% A (water with 5% DMSO, 0.1% formic acid) to 65% A/35% B (acetonitrile with 5% DMSO, 0.1% formic acid) in 60 or 120 minutes. Eluting peptides were ionized in the positive ion mode. One survey scan was performed in the Orbitrap part of the instrument with a mass range of m/z 400-1200 and a resolution of 60,000 followed by fragmentation of the 10 most abundant signals in the ion trap part of the instrument. The dynamic exclusion was set to 60 seconds and the repeat count to 1. Raw files were processed using Maxquant[18] as described previously[16] with the following settings: precursor ion tolerance for the first search: 20 ppm; fragment ion tolerance: 0.5 Da; fixed modification: propionamide at cystein; variable modifications: oxidation at methionine, acetylation at protein N-termini; protease: trypsin; maximum number of missed cleavage sites: 2; database: SwissProt/TREMBL (79,899 entries, release date: 09/2016, MaxQuant Version: 1.5.1.0). Data were further analyzed using MS Excel.

All raw and processed data are available upon request.

SUPPLEMENTARY FIGURES

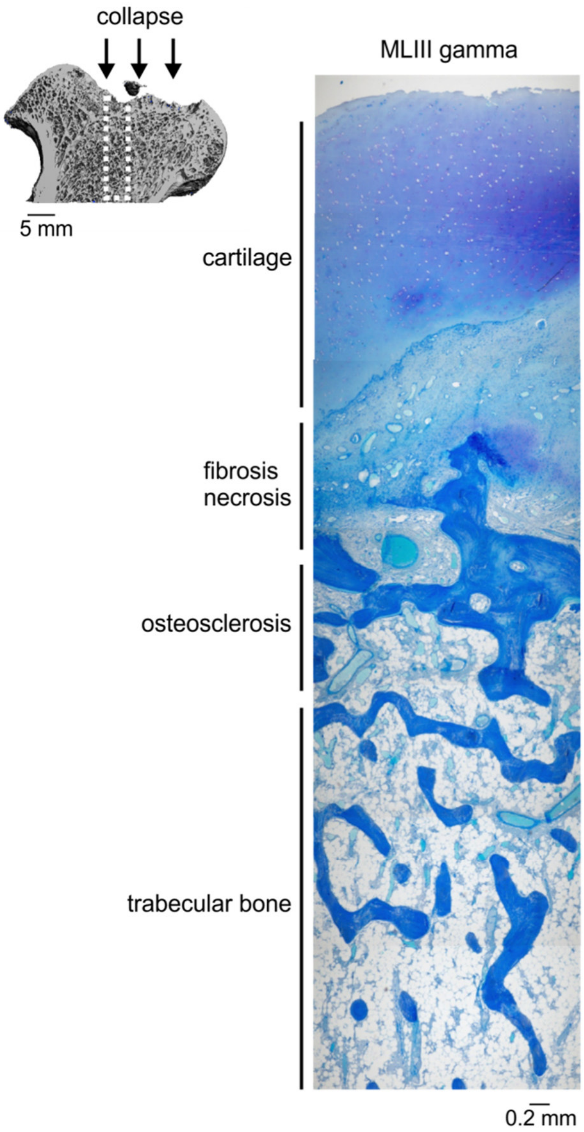

Fig. S1 Histological imaging of the collapsed femoral head of patient #1 with MLIII gamma. White dotted box from high-resolution CT of the femoral head (upper left image) indicates the region of interest for histology (toluidine blue staining). From top to bottom, the collapsed femoral head shows remaining cartilage, signs of necrosis and fibrosis and underlying osteosclerosis. Underneath the osteonecrosis area (bottom region), trabecular bone displaying high bone turnover is found.

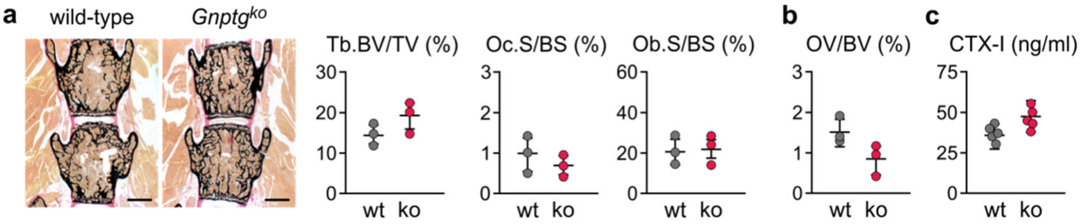

Fig. S2 No bone alterations in 45-week-old *Gnptg*^{ko} mice. (**a**) Representative von Kossa/van Gieson staining of non-decalcified vertebra sections from wild-type (wt) and *Gnptg*^{ko} (ko) mice (scale bars: 1 mm). Quantification of the trabecular bone volume per tissue volume (Tb.BV/TV), osteoclast surface per bone surface (Oc.S/BS) and osteoblast surface per bone surface (Ob.S/BS) of the same mice is given on the right (n = 3, mean ± SD). (**b**) Quantification of the vertebral osteoid volume per bone volume (OV/BV) in wt and *Gnptg*^{ko} (ko) mice (n = 3, mean ± SD). (**c**) C-terminal telopeptides of type I collagen (CTX-I) in wt and *Gnptg*^{ko} (ko) mice (n = 5, mean ± SD).

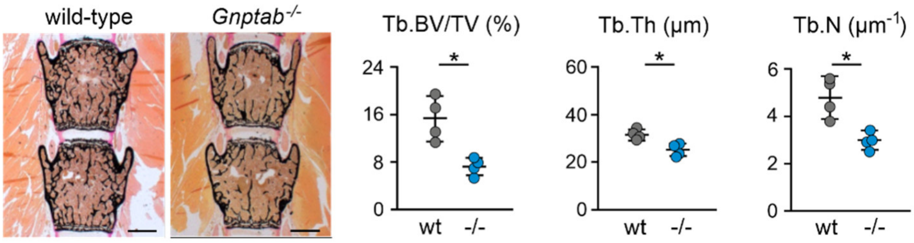

Fig. S3 Low bone mass in 12-week-old *Gnptab*$^{-/-}$ mice. (**a**) Representative von Kossa/van Gieson staining of non-decalcified vertebra sections from wild-type (wt) and *Gnptab*$^{-/-}$ mice (scale bars: 1 mm). Quantification of the trabecular bone volume per tissue volume (Tb.BV/TV), trabecular thickness (Tb.Th) and trabecular number (Tb.N) from the same mice are given on the right (n = 4, mean ± SD).

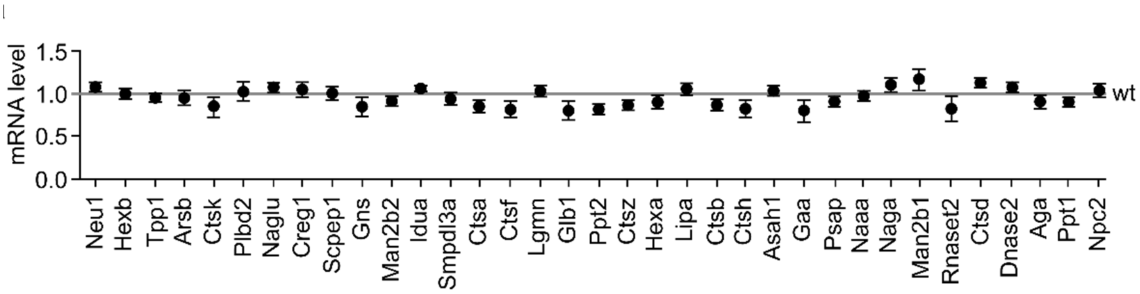

Fig. S4 Transcript levels of lysosomal enzymes. Relative mRNA expression levels of indicated genes encoding soluble lysosomal proteins were determined by qPCR analysis of wild-type (wt = 1.0, grey line) and *Gnptg*^{ko} osteoblasts (d25) (n = 3).

SUPPLEMENTARY TABLES

Table S1: Molecular diagnosis and urinary Dpd/Crea ratios of patients with MLIII gamma, MLIII alpha/beta and MLII.

Nr	Sex	Age[a]	Disease	Molecular diagnosis	Origin, Reference[b]	Dpd/Crea nmol/mmol
1	F	14	MLIII gamma[c]	*GNPTG* c.499dup/c.499dup	Iraq, this study	15
2	M	6	MLIII gamma[d]	*GNPTG* c.499dup/c.499dup	Iraq, this study	25
3	M	7	MLIII gamma	*GNPTG* c.527-10G>A/c.527-10G>A	Afghanistan[19]	5
4	M	21	MLIII gamma	*GNPTG* c.527-10G>A/c.527-10G>A	Afghanistan[19]	4
5	F	22	MLIII gamma	*GNPTG* c.619_620insT/c.619_620insT	Germany[20]	6
6	M	46	MLIII gamma	*GNPTG* c.328G>T/c.328G>T	Brazil[21]	5
7	M	49	MLIII gamma	*GNPTG* c.328G>T/c.328G>T	Brazil[21]	5
8	M	16	MLIII alpha/beta[e]	*GNPTAB* c.2591_2592insG/c.3335+6T>G	UK, this study	n.d.
9	M	6	MLIII alpha/beta	*GNPTAB* c.1196C>T/c.2757_2758del	Brazil, this study	41
10	M	11	MLIII alpha/beta[f]	*GNPTAB* c.10A>C/c.2502del	Germany[22]	24
11	M	12	MLIII alpha/beta	*GNPTAB* c.3503_3504del/c.?	Brazil[10]	26
12	F	13	MLIII alpha/beta	*GNPTAB* c.1208T>C/c.832C>T	Brazil[13]	35
13	F	21	MLIII alpha/beta	*GNPTAB* c.3503_3504del/c.1208T>C	Brazil[13]	16
14	M	34	MLIII alpha/beta	*GNPTAB* c.3503_3504del/c.1514G>A	Brazil[13]	9
15	M	0.7	MLII	*GNPTAB* c.2249dup/c.2249dup	Brazil, this study	61
16	F	2	MLII	*GNPTAB* c.344_345del/c.1022del	Germany[3]	50
17	F	4	MLII	*GNPTAB* c.2213C>A/c.2220_2221dup	Austria[23]	43
C-1	F	9	control		Germany, this study	14
C-2	F	13	control		Germany, this study	12
C-3	F	16	control		Germany, this study	13
C-4	F	23	control		Germany, this study	5
C-5	M	25	control		Germany, this study	4
C-6	F	29	control		Germany, this study	5
C-7	F	43	control		Germany, this study	6
C-8	F	44	control		Germany, this study	5
C-9	M	50	control		Germany, this study	3

[a]age in years at the moment of taking urine samples, [b]reference for molecular and/or clinical diagnosis, [c]femoral head biopsy taken at the age of 16 years (see Fig. 1c), [d]iliac crest biopsy taken at the age of 8 years (see Fig. 1d),[e]iliac crest biopsy taken at the age of 16 years (see Fig. 1e), [f]clinically this patient represents an intermediate form between MLII and MLIII alpha/beta.

Table S2: Parameters of bone histomorphometry in iliac crest biopsies from patient #2 (MLIII gamma) and patient #8 (MLIII alpha/beta) compared to age-matched control biopsies (mean ± SD).

**Parameter**	**MLIII gamma** Patient #2	**Reference**[24] (n = 6)	**MLIII alpha/beta** Patient #8	**Reference**[24] (n = 10)
Age	8	2-8	16	18-25
BV/TV (%)	20.01	17.82 ± 7.20	16.89	22.16 ± 5.66
Tb.Th (mm)	89.59	105.2 ± 17.5	142.4	158.2 ± 33.8
Tb.N (mm^{-1})	2.23	1.66 ± 0.50	1.18	1.40 ± 0.21
Tb.Sp (mm)	358.1	557.4 ± 261.9	700.49	570.0 ± 114.7
OS/BS (%)	19.68	15.76 ± 11.49	25.86	16.58 ± 11.36
Ob.S/BS (%)	18.72	25.64 ± 9.97	9.57	14.73 ± 5.09
Oc.S/BS (%)	1.56	1.92 ± 2.28	1.96	0.47 ± 0.53

BV/TV: bone volume per tissue volume, Tb.Th: trabecular thickness, Tb.N: trabecular number, Tb.Sp: trabecular space, OS/BS: osteoid surface/bone surface, Ob.S/BS: osteoblast surface per bone surface, Oc.S/BS: osteoclast surface per bone surface.

Table S3. Lysosomal soluble proteins identified in bound fractions by M6P antibody affinity chromatography of wild-type and *Gnptg*^{ko} osteoblasts in three replicates.

Uniprot ID	Protein	Gene	ko/wt ratio		
			mean	SD	P-value
O35657	sialidase 1	*Neu1*	0.21	0.01	0.013
P20060	β-hexosaminidase β-subunit	*Hexb*	0.25	0.04	0.003
O89023	tripeptidyl peptidase I	*Tpp1*	0.25	0.01	0.015
P50429	arylsulfatase B	*Arsb*	0.25	0.03	0.015
P55097	cathepsin K	*Ctsk*	0.26	0.09	0.004
Q3TCN2	putative phospholipase B-like 2	*Plbd2*	0.27	0.04	0.017
O54752	N-acetyl-α-glucosaminidase	*Naglu*	0.30	0.02	0.018
O88668	Creg1 protein	*Creg1*	0.31	0.06	0.020
Q920A5	serine carboxypeptidase 1	*Scpep1*	0.33	0.02	0.020
Q8BFR4	N-acetylglucosamine-6-sulfatase	*Gns*	0.34	0.03	0.022
O54782	α-mannosidase, class 2B member 2	*Man2b2*	0.35	0.06	0.023
P48441	α-iduronidase	*Idua*	0.36	0.02	0.023
P70158	acid sphingomyelinase-like phosphodiesterase 3a	*Smpdl3a*	0.36	0.11	0.028
P16675	cathepsin A	*Ctsa*	0.38	0.06	0.027
Q9R013	cathepsin F	*Ctsf*	0.40	0.01	0.028
O89017	legumain	*Lgmn*	0.40	0.07	0.030
P23780	β-galactosidase	*Glb1*	0.41	0.05	0.029
O35448	palmitoyl-protein thioesterase 2	*Ppt2*	0.41	0.11	0.035
Q9WUU7	cathepsin Z	*Ctsz*	0.43	0.08	0.035
P29416	β-hexosaminidase α-subunit	*Hexa*	0.51	0.02	0.014
Q9Z0M5	lysosomal acid lipase	*Lipa*	0.52	0.10	0.020
P10605	cathepsin B	*Ctsb*	0.52	0.14	0.064
P49935	cathepsin H	*Ctsh*	0.54	0.07	0.058
Q9WV54	acid ceramidase	*Asah1*	0.57	0.32	0.148
P70699	α-glucosidase	*Gaa*	0.61	0.15	0.053
Q61207	prosaposin	*Psap*	0.67	0.04	0.112
Q9D7V9	N-acylethanolamine acid amidase	*Naaa*	0.81	0.11	0.308
Q9QWR8	α-N-acetylgalactosaminidase	*Naga*	0.82	0.06	0.313
O09159	α-mannosidase, class 2B member 1	*Man2b1*	0.83	0.21	0.412
Q9CQ01	ribonuclease T2	*Rnaset2*	0.83	0.46	0.582
P18242	cathepsin D	*Ctsd*	0.87	0.23	0.498
P56542	deoxyribonuclease II	*Dnase2*	0.97	0.37	0.917
Q64191	aspartylglucosaminidase	*Aga*	1.08	0.33	0.748
O88531	palmitoyl-protein thioesterase 1	*Ppt1*	1.18	0.05	0.331
Q9Z0J0	Niemann-Pick disease type C2 protein	*Npc2*	1.20	0.21	0.449

SUPPLEMENTARY REFERENCES

1. Raas-Rothschild A, Cormier-Daire V, Bao M et al. Molecular basis of variant pseudo-hurler polydystrophy (mucolipidosis IIIC). J Clin Invest. 2000;105:673-681.
2. Tüysüz B, Kasapcopur O, Alkaya DU, Sahin S, Sozeri B, Yesil G. Mucolipidosis type III gamma: Three novel mutation and genotype-phenotype study in eleven patients. Gene. 2018;642:398-407.
3. Velho RV, Harms FL, Danyukova T et al. The lysosomal storage disorders mucolipidosis type II, type III alpha/beta and type III gamma: Update on GNPTAB and GNPTG mutations. Hum Mutat. 2019;40:842-864.
4. Cathey SS, Leroy JG, Wood T et al. Phenotype and genotype in mucolipidoses II and III alpha/beta: a study of 61 probands. J Med Genet. 2010;47:38-48.
5. Bargal R, Zeigler M, Abu-Libdeh B et al. When mucolipidosis III meets mucolipidosis II: GNPTA gene mutations in 24 patients. Mol Genet Metab. 2006;88:359-363.
6. David-Vizcarra G, Briody J, Ault J et al. The natural history and osteodystrophy of mucolipidosis types II and III. J Paediatr Child Health. 2010;46:316-322.
7. Kudo M, Canfield WM. Structural requirements for efficient processing and activation of recombinant human UDP-N-acetylglucosamine:lysosomal-enzyme-N-acetylglucosamine-1-phosphotransferase. J Biol Chem. 2006;281:11761-11768.
8. Leroy JG, Sillence D, Wood T et al. A novel intermediate mucolipidosis II/IIIalphabeta caused by GNPTAB mutation in the cytosolic N-terminal domain. Eur J Hum Genet. 2014;22:594-601.
9. Coutinho MF, Encarnacao M, Laranjeira F, Lacerda L, Prata MJ, Alves S. Solving a case of allelic dropout in the GNPTAB gene: implications in the molecular diagnosis of mucolipidosis type III alpha/beta. J Pediatr Endocrinol Metab. 2016;29:1225-1228.
10. Cury GK, Matte U, Artigalas O et al. Mucolipidosis II and III alpha/beta in Brazil: Analysis of the GNPTAB gene. Gene. 2013;524:59-64.
11. Encarnação M, Lacerda L, R. C et al. Molecular analysis of the GNPTAB and GNPTG genes in 13 patients with mucolipidosis type II or type III - identification of eight novel mutations. Clin. Genet. 2009;76:76-84.
12. Gheldof A, Seneca S, Stouffs K et al. Clinical implementation of gene panel testing for lysosomal storage diseases. Mol Genet Genomic Med. 2019;7:e00527.
13. Ludwig NF, Velho RV, Sperb-Ludwig F et al. GNPTAB missense mutations cause loss of GlcNAc-1-phosphotransferase activity in mucolipidosis type II through distinct mechanisms. Int J Biochem Cell Biol. 2017;92:90-94.
14. Schulze J, Bickert T, Beil FT et al. Interleukin-33 is expressed in differentiated osteoblasts and blocks osteoclast formation from bone marrow precursor cells. J Bone Miner Res. 2011;26:704-717.
15. Dempster DW, Compston JE, Drezner MK et al. Standardized nomenclature, symbols, and units for bone histomorphometry: a 2012 update of the report of the ASBMR Histomorphometry Nomenclature Committee. J Bone Miner Res. 2013;28:2-17.
16. Di Lorenzo G, Velho RV, Winter D et al. Lysosomal proteome and secretome analysis identifies missorted enzymes and their non-degraded substrates in mucolipidosis III mouse cells. Mol Cell Proteomics. 2018;17:1612-1626.
17. Rappsilber J, Mann M, Ishihama Y. Protocol for micro-purification, enrichment, pre-fractionation and storage of peptides for proteomics using StageTips. Nat Protoc. 2007;2:1896-1906.
18. Cox J, Mann M. MaxQuant enables high peptide identification rates, individualized p.p.b.-range mass accuracies and proteome-wide protein quantification. Nat Biotechnol. 2008;26:1367-1372.
19. Pohl S, Encarnação M, Castrichini M, Müller-Loennies S, Muschol N, Braulke T. Loss of N-Acetylglucosamine-1-phosphotransferase gamma-subunit due to intronic mutation in GNPTG causes mucolipidosis type III gamma: Implications for molecular and cellular diagnostics. Am J Med Genet A. 2010;152A:124–132.

20. Pohl S, Tiede S, Castrichini M, Cantz M, Gieselmann V, Braulke T. Compensatory expression of human N-Acetylglucosaminyl-1-phosphotransferase subunits in mucolipidosis type III gamma. Biochim Biophys Acta. 2009;1792:221-225.
21. Velho RV, Ludwig NF, Alegra T et al. Enigmatic in vivo GlcNAc-1-phosphotransferase (GNPTG) transcript correction to wild type in two mucolipidosis III gamma siblings homozygous for nonsense mutations. J Hum Genet. 2016;61:555-560.
22. Ammer LS, Oussoren E, Muschol N et al. Hip morphology in mucolipidosis type II. J Clin Med. 2020;9:728.
23. Köhne T, Markmann S, Schweizer M et al. Mannose 6-phosphate-dependent targeting of lysosomal enzymes is required for normal craniofacial and dental development. Biochim Biophys Acta. 2016;1862:1570-1580.
24. Jandl NM, von Kroge S, Stürznickel J et al. Large osteocyte lacunae in iliac crest infantile bone are not associated with impaired mineral distribution or signs of osteocytic osteolysis. Bone. 2020;135:115324.
